# Supplementary material for: The genome sequence of a false flower beetle, Anaspis maculata (Geoffroy in Fourcroy, 1785)
Source: Wellcome Open Res. 2024 Apr 23;9:212. [Version 1] doi: 10.12688/wellcomeopenres.21283.1 (PMC11252652; doi:10.12688/wellcomeopenres.21283.1)
Supplement: Peer review history

# Reviewer report: Reviewer response for version 1

**DOI:** 10.21956/wellcomeopenres.23539.r88821

**Published:** 2024-07-16

**Authors:** Phillip Davidson

**Affiliations:** 1 Department of Biology, Indiana University Bloomington, Bloomington, Indiana, USA

In their genome report, Telfer *et al*., present a complete and high quality reference assembly for the false flower beetle *Anaspis maculata. *The rationale, methodology, and data availability are sound and appropriate for the study.  I have a handful of revision suggestions that I think will clarify the results of this data set, as listed below. 

 Specific comments:

- Typo: **Background** line 5: remove “of”.
- Run-on sentence: **Background** line 9: change “scavengers, they” to “scavengers. They”.
- In methods and results, please specify what developmental stage the specimen used for sequencing was (adult, pupa, larva).
- Figure 2, BUSCO miniplot: The percentages displayed add up to greater than 100%, suggesting an error in the labelling. The middle shade of green I think represents single copy, not complete, composition. Please check and if this true, an accurate plot and labelled should be supplied.
- Please provide a scale bar for Figure 1, as this is standard for presenting specimen images in genome reports. 

Are sufficient details of methods and materials provided to allow replication by others?

Yes

Is the rationale for creating the dataset(s) clearly described?

Yes

Are the datasets clearly presented in a useable and accessible format?

Yes

Are the protocols appropriate and is the work technically sound?

Yes

Reviewer Expertise:

Genome assembly and annotation, comparative genomics, evo-devo

I confirm that I have read this submission and believe that I have an appropriate level of expertise to confirm that it is of an acceptable scientific standard, however I have significant reservations, as outlined above.

# Reviewer report: Reviewer response for version 1

**DOI:** 10.21956/wellcomeopenres.23539.r88822

**Published:** 2024-07-16

**Authors:** José María Martín-Durán

**Affiliations:** 1 Queen Mary University of London, London, UK

This genome note reports the assembly and annotation of the genome of the beetle *Anaspis maculata*. This is a common species in the UK and most parts of Western Europe. This genome will help investigate the diversity of Coleoptera and contribute to ecological and evolutionary studies.

 Strengths of the resource:

- High quality assembly, annotated following the high standards of the Darwin Tree of Life.
- Methods and approaches are well described.

Are sufficient details of methods and materials provided to allow replication by others?

Yes

Is the rationale for creating the dataset(s) clearly described?

Yes

Are the datasets clearly presented in a useable and accessible format?

Yes

Are the protocols appropriate and is the work technically sound?

Yes

Reviewer Expertise:

Evolutionary biology, comparative genomics

I confirm that I have read this submission and believe that I have an appropriate level of expertise to confirm that it is of an acceptable scientific standard.

# Reviewer report: Reviewer response for version 1

**DOI:** 10.21956/wellcomeopenres.23539.r88825

**Published:** 2024-07-09

**Authors:** Doga Cedden

**Affiliations:** 1 Department of Evolutionary Developmental Genetics, Johann-Friedrich-Blumenbach Institute, Göttingen, Germany

Data note by Telfer *et al.* presents a high-quality genome assembly from *Anaspis maculata*. The background information about this saproxylic beetle is adequately covered in the data note. The method is sufficiently described and appropriate, and the authors also present a sufficient quality assessment of the assembly. Below are several comments for consideration:

1. False flower beetle may refer to *Scraptiidae* or sometimes specifically to the genus *Anaspis* but not specifically to *Anaspis maculata*. This is appropriately indicated in the title by "a false flower beetle." Could the authors also indicate this in the abstract? Rather than "false flower beetle," it could be referred to as "a member of a false flower beetle" or "a false flower beetle" in parentheses.
2. It would have been better to provide some kind of scale in Figure 1, which is commonly done in other submissions by the Darwin Tree of Life Project. Please consider this in future submissions.
3. Could the authors check the GC% provided in Table 2? I found it a bit surprising that all autosomal chromosomes had a "40.0%" GC content. It could also be the actual values, which is totally fine.

Are sufficient details of methods and materials provided to allow replication by others?

Yes

Is the rationale for creating the dataset(s) clearly described?

Yes

Are the datasets clearly presented in a useable and accessible format?

Yes

Are the protocols appropriate and is the work technically sound?

Yes

Reviewer Expertise:

I work on coleopteran pests using RNA interference and RNA-seq.

I confirm that I have read this submission and believe that I have an appropriate level of expertise to confirm that it is of an acceptable scientific standard.
